# Extraction of Teeth without the Lancet

**Published:** 1865-11

**Authors:** R. J. Poore

**Affiliations:** Lexington, Ky.


THE
DENTAL REGISTER OF THE WEST.

VOL. XIX.] NOVEMBER, 1865. [No. 11.

### Original Essays and Communications.

### EXTRACTION OF TEETH WITHOUT THE LANCET.

BY R. J. POORE.

In the number of the *Register* for May and June I read a Preamble and Resolution passed by the Louisville Dental Association, eminating from a discussion of its members upon the utility of lancing the gums, preparatory to the extraction of teeth; and as to the propriety of the use of water after the operation, with a request that societies and practitioners generally, make report of their practice and experience upon the same.

I have looked with no little solicitude for a response from some one, but have thus far been disappointed. The reticence of the advocates of the *inutility* of the use of the gum-lancet, may proceed, perhaps from a delicacy to shoulder responsibility—to avoid the criticisms of those of the profession who are *set in their ways.* But, as I believe a revolution is destined to occur in the practice under controversy, as soon as old, stubborn prejudices can be broken down—as soon as the profession can be convinced of the practicability of the method, and, as I have for the last fifteen years almost entirely dispensed with the use of the gum-lancet, I feel myself called upon to give my testimony, and I shall do so, regardless of whatever criticisms may be made, knowing that *good*, results from investigation, and that *enlightenment* follows controversy. It is to be expected, that upon the introduction of "something new," or, a report upon some case of "heroic" practice, you will collide with those who nurture prejudices in favor of the old "court system, or practice," who cling to their old ways with a stubborn pertinacity, until popular favor demands, both in and out of the profession, a surrender.

The inutility of the general use of the gum-lancet has been recognized and practiced by many of the profession for years. I have been surprised that some one has not communicated his faith and practice long since, but they have been like myself, *modest* and negligent. I hope the accusation of *modesty* will not be chargable to the profession upon the "*gum-tearing*" subject any longer.

That I may give a clearer idea of the subject, I will report my induction, into the method under discussion, I received my first lesson practically, from the great, the lamented Dr. S. P. Hullihen, of Wheeling, Va. Receiving a *gentle reminder* from an *old sentenal*, I rode forty miles for the best skill, to have it removed, (for be it known a tooth-carpenter is as averse to being hurt as any other man) as the Dr's. reputation in that speciality, particularly extended throughout the country. When I reported myself, *sans ceremonie*, he told me to take the chair, asked which tooth. I opened my mouth *wide*, supposed he would use the inevitable lancet of course, braced myself, but to my great *happification* in an instant he showed me the villainous offender, without giving me, I can honestly say, the pain I apprehended, and had experienced, from the *hacking* and *gouging* of the gum lancet. I then learned one of the secrets of his enviable success, and reputation. Inquired if he never used the lancet, "no sir, rarely ever, only in some peculiar case, not once in a hundred instances, it is an unnecessary infliction sir." I was a convert to the new system at once — availed myself of the priveleges of further light, under his courteous and skillful instruction, and have practiced it ever since, with entire satisfaction to myself, and have been assured in all instances by my patients, that it was equally so to them. The few of the profession with whom I have conversed upon this subject, I was satisfied, thought me a *hobby rider*, but I disclaim the charge. Yet, call the practice what you please, I am conscious of this fact, that I have rode it into the mouths of many, very many, in which I would not have had the pleasure (!) of manipulating, had it not been for said "*hobby ?*"— prepared mouths for timid persons, for sets of teeth, that would not have submitted to the operation, had I used the lancet. The complaints as you are aware, from a majority of patients, are against cutting the gums, the unpleasant recollections after the operation are, the *cutting*, *hacking* and *gouging* around the gums. The use of the gum lancet is a preparatory step to an apprehended greater pain. The patients mind is filled with fear and excitement—all the sensibilities, consequently are intensified to the highest degree of susceptibility to pain — in fact he is placed in just that condition to make him realize *all* the pain attendant upon the operation, whereas, *sans ceremonie*, the displacement of the gums with the forceps, and the simultaneous removal of the tooth by an expert operator, takes the patient so by surprise, that he rises out of your chair declaring that he suffered very little, or no pain at all. Surely, such results should be sought after by every humane operator, particularly when no compromise is made with what I contend to be a *scientific* course of practice.

It is the duty too of every dentist, to resort to all the *arts* of which he is capable, to lighten the inflictions of his patient, particularly so, while he is under the ordeal of parting with several of his grinders. A great deal therefore depends upon the *manner of the operator*, under such circumstances; as it is not the intention of this paper to occupy space upon that subject, I will but say, that it is presumption — that every dentist is fully sensible of the importance, of *preparing* his patient for the intended operation, whatever it may be. If he is not, he should make those matters a subject of close study and qualify himself for all exigencies. My forceps are well adapted to the teeth, not, however, unlike those generally in use by first class operators. If your forceps are well adapted to the teeth, then all depends, as my preceptor impressed upon me, *upon the manner of using them.* Place your forceps, said he, properly upon the tooth, then shove it home like you were trying to push the tooth through the jaw, you then have seperated the gum from the tooth, got a good secure hold, and but little force comparatively is necessary to complete the operation. By obeying this injunction my difficulties have been comparatively few, rarely failing to remove the tooth the first effort. The fear of getting too high a hold, is the cause of many otherwise good operators, being mortified with frequent failures in the extraction of shelly teeth. But to the subject. In all my experience in this practice, I have torn the gums in but very few instances, and they would have occurred had the lancet been used, for they were cases of alveolar attachment, or, osseous union, when such result would have been unavoidable. It is important under all circumstances that the operator should keep his eye closely upon the tooth, so that in the event of an attachment of the kind he can timely avert unnecessary laceration or tearing. I have taken particular pains in making examinations; and have universally found that the gums heal as rapidly as if carefully cut with a sharp lancet. In fact, I believe the operation to say the least, quite as neatly done with the forceps, properly applied, as it can be in the large majority of cases with the lancet, and the hemorrhage after the operation is no greater, if really as great. So, consequently, I take the position, notwithstanding I have Taft and all other authors on operative dentistry against me, and give it as my unhesitating conviction, that the gum-lancet is altogether unnecessary except in extreme cases. If a fang is to be extracted, which is concealed under the gums, I use the punch and "go for it," as I do with the forceps for a tooth. This method or practice condenses the operation into one *thrust* and *pull*, making it more expeditious, less troublesome to the operator, and vastly more agreeable to the patient. It is preferable too, from the fact, you will succeed in very many instances when you would not, if you attempted to use the lancet, particularly with children. Am satisfied, therefore, if the practice is adopted by those of the profession, *who can possess themselves*—can keep cool—that a'nt bothered with the shakes, I repeat, *can possess themselves*, so that they can watch closely what they are doing, when pulling a tooth, they will rarely have use for the gum-lancet again.

As to the use of water after the operation, I will but report my practice. I permit the patient to use all the water they wish, after the hemorrhage has ceased. I touch the gums with Arnica and Camphor, (two parts Arnica and one Camphor,) and recommend the patient to use the remedy twice or three times a day, until the gums are pretty well healed. I find it most excellent in curing up the gums and keeping them from soreness.

LEXINGTON, KY.

